# Supplementary material for: Ordinal Outcome State-Space Models for Intensive Longitudinal Data
Source: Psychometrika. 2024 Jun 11;89(4):1203–29. doi: 10.1007/s11336-024-09984-3 (PMC11582181; doi:10.1007/s11336-024-09984-3)
Supplement: Supplementary file 1 — (pdf 263 KB) [file 11336_2024_9984_MOESM1_ESM.pdf]

SUPPLEMENTARY MATERIALS FOR "ORDINAL OUTCOME STATE-SPACE MODELS FOR INTENSIVE LONGITUDINAL DATA"

1. Result Tables

Table S1.
State Recovery and Autoregressive Parameter Outcomes for 100 Timepoint Conditions. N Items: Number of Items per State; N Responses: Number of Response Categories per Item.

AR/CR Parameters	Spread	N Items	N Responses	State Recovery		Median Relative Bias		Slice SE		Coverage	
				GRM	Linear	GRM	Linear	GRM	Linear	GRM	Linear
AR: 0.3 / CR: 0	Equal	3	3	0.647	0.528	-0.142	0.971	0.169	0.108	0.838	0.361
AR: 0.3 / CR: 0	Equal	3	7	0.681	0.579	-0.186	0.987	0.163	0.093	0.765	0.287
AR: 0.3 / CR: 0	Equal	6	3	0.769	0.675	-0.072	0.221	0.122	0.277	0.796	0.577
AR: 0.3 / CR: 0	Equal	6	7	0.8	0.755	0.06	0.884	0.122	0.192	0.778	0.472
AR: 0.3 / CR: 0	Offset	3	3	0.625	0.474	-0.158	0.939	0.183	0.114	0.765	0.4
AR: 0.3 / CR: 0	Offset	3	7	0.68	0.572	-0.173	0.971	0.167	0.091	0.807	0.29
AR: 0.3 / CR: 0	Offset	6	3	0.695	0.411	-0.128	-0.152	0.152	0.253	0.808	0.412
AR: 0.3 / CR: 0	Offset	6	7	0.789	0.669	0.005	0.61	0.114	0.239	0.746	0.574
AR: 0.3 / CR: 0.25	Equal	3	3	0.657	0.564	-0.091	0.806	0.174	0.111	0.788	0.441
AR: 0.3 / CR: 0.25	Equal	3	7	0.69	0.61	-0.078	0.801	0.165	0.095	0.816	0.373
AR: 0.3 / CR: 0.25	Equal	6	3	0.776	0.706	-0.102	0.234	0.118	0.26	0.794	0.577
AR: 0.3 / CR: 0.25	Equal	6	7	0.803	0.764	0.047	0.735	0.122	0.183	0.772	0.522
AR: 0.3 / CR: 0.25	Offset	3	3	0.625	0.491	-0.247	0.765	0.181	0.125	0.766	0.49
AR: 0.3 / CR: 0.25	Offset	3	7	0.69	0.594	-0.098	0.827	0.171	0.098	0.835	0.397
AR: 0.3 / CR: 0.25	Offset	6	3	0.698	0.419	-0.137	-0.238	0.151	0.256	0.769	0.399
AR: 0.3 / CR: 0.25	Offset	6	7	0.797	0.688	0	0.519	0.115	0.242	0.749	0.59
AR: 0.7 / CR: 0	Equal	3	3	0.691	0.63	-0.143	0.043	0.131	0.076	0.767	0.632
AR: 0.7 / CR: 0	Equal	3	7	0.731	0.666	-0.083	0.074	0.122	0.062	0.775	0.535
AR: 0.7 / CR: 0	Equal	6	3	0.796	0.696	-0.064	-0.34	0.094	0.257	0.802	0.615
AR: 0.7 / CR: 0	Equal	6	7	0.826	0.785	-0.009	0	0.083	0.161	0.786	0.59
AR: 0.7 / CR: 0	Offset	3	3	0.671	0.568	-0.139	-0.007	0.14	0.094	0.782	0.648
AR: 0.7 / CR: 0	Offset	3	7	0.724	0.664	-0.119	0.079	0.127	0.063	0.794	0.535
AR: 0.7 / CR: 0	Offset	6	3	0.737	0.443	-0.099	-0.671	0.114	0.226	0.766	0.332
AR: 0.7 / CR: 0	Offset	6	7	0.808	0.714	-0.024	-0.2	0.083	0.226	0.758	0.674
AR: 0.7 / CR: 0.25	Equal	3	3	0.718	0.666	-0.122	-0.113	0.14	0.113	0.785	0.712
AR: 0.7 / CR: 0.25	Equal	3	7	0.752	0.706	-0.091	-0.079	0.139	0.086	0.8	0.627
AR: 0.7 / CR: 0.25	Equal	6	3	0.81	0.732	-0.05	-0.413	0.094	0.303	0.768	0.621
AR: 0.7 / CR: 0.25	Equal	6	7	0.833	0.788	-0.021	-0.168	0.092	0.213	0.773	0.721
AR: 0.7 / CR: 0.25	Offset	3	3	0.693	0.616	-0.129	-0.148	0.154	0.116	0.787	0.681
AR: 0.7 / CR: 0.25	Offset	3	7	0.751	0.699	-0.09	-0.067	0.134	0.094	0.828	0.642
AR: 0.7 / CR: 0.25	Offset	6	3	0.755	0.497	-0.105	-0.644	0.121	0.248	0.762	0.358
AR: 0.7 / CR: 0.25	Offset	6	7	0.828	0.739	-0.034	-0.314	0.09	0.247	0.78	0.715

Table S2.
State Recovery and Autoregressive Parameter Outcomes for 500 Timepoint Conditions. N Items: Number of Items per State; N Responses: Number of Response Categories per Item.

AR/CR Parameters	Spread	N Items	N Response	State Recovery		Median Rel. Bias		Median Slice SE		Coverage	
				GRM	Linear	GRM	Linear	GRM	Linear	GRM	Linear
AR: 0.3 / CR: 0	Equal	3	3	0.658	0.482	-0.073	1.16	0.067	0.046	0.77	0.091
AR: 0.3 / CR: 0	Equal	3	7	0.698	0.573	-0.046	1.183	0.063	0.038	0.747	0.04
AR: 0.3 / CR: 0	Equal	6	3	0.777	0.593	0	-0.139	0.047	0.089	0.714	0.207
AR: 0.3 / CR: 0	Equal	6	7	0.807	0.738	0.015	0.469	0.046	0.085	0.677	0.323
AR: 0.3 / CR: 0	Offset	3	3	0.635	0.417	-0.028	1.109	0.073	0.047	0.784	0.088
AR: 0.3 / CR: 0	Offset	3	7	0.697	0.558	-0.024	1.199	0.062	0.038	0.77	0.038
AR: 0.3 / CR: 0	Offset	6	3	0.707	0.354	-0.03	-0.168	0.059	0.078	0.718	0
AR: 0.3 / CR: 0	Offset	6	7	0.798	0.637	0.051	0.248	0.044	0.099	0.63	0.357
AR: 0.3 / CR: 0.25	Equal	3	3	0.663	0.527	-0.054	0.971	0.068	0.049	0.778	0.135
AR: 0.3 / CR: 0.25	Equal	3	7	0.7	0.603	-0.033	0.912	0.062	0.041	0.763	0.128
AR: 0.3 / CR: 0.25	Equal	6	3	0.78	0.631	0.01	-0.022	0.047	0.094	0.71	0.244
AR: 0.3 / CR: 0.25	Equal	6	7	0.81	0.744	0.045	0.311	0.046	0.085	0.654	0.324
AR: 0.3 / CR: 0.25	Offset	3	3	0.642	0.453	-0.025	0.904	0.073	0.05	0.747	0.174
AR: 0.3 / CR: 0.25	Offset	3	7	0.704	0.602	-0.002	0.959	0.062	0.043	0.766	0.129
AR: 0.3 / CR: 0.25	Offset	6	3	0.71	0.37	-0.05	-0.302	0.057	0.077	0.733	0
AR: 0.3 / CR: 0.25	Offset	6	7	0.801	0.655	0.028	0.042	0.045	0.095	0.68	0.309
AR: 0.7 / CR: 0	Equal	3	3	0.717	0.661	-0.028	0.182	0.046	0.024	0.728	0.168
AR: 0.7 / CR: 0	Equal	3	7	0.748	0.702	-0.012	0.188	0.042	0.019	0.736	0.109
AR: 0.7 / CR: 0	Equal	6	3	0.81	0.635	-0.019	-0.475	0.034	0.089	0.718	0
AR: 0.7 / CR: 0	Equal	6	7	0.836	0.767	0.016	-0.148	0.03	0.087	0.63	0.255
AR: 0.7 / CR: 0	Offset	3	3	0.697	0.582	-0.033	0.147	0.049	0.027	0.767	0.262
AR: 0.7 / CR: 0	Offset	3	7	0.748	0.701	-0.019	0.191	0.043	0.019	0.732	0.112
AR: 0.7 / CR: 0	Offset	6	3	0.755	0.398	-0.02	-0.694	0.041	0.081	0.761	0.097
AR: 0.7 / CR: 0	Offset	6	7	0.826	0.677	0.012	-0.366	0.031	0.099	0.646	0.294
AR: 0.7 / CR: 0.25	Equal	3	3	0.743	0.689	-0.038	-0.004	0.05	0.038	0.737	0.5
AR: 0.7 / CR: 0.25	Equal	3	7	0.773	0.738	-0.041	0.034	0.047	0.031	0.719	0.48
AR: 0.7 / CR: 0.25	Equal	6	3	0.823	0.681	-0.012	-0.554	0.035	0.097	0.72	0.158
AR: 0.7 / CR: 0.25	Equal	6	7	0.849	0.79	0.003	-0.312	0.032	0.092	0.671	0.287
AR: 0.7 / CR: 0.25	Offset	3	3	0.73	0.63	-0.039	-0.033	0.053	0.039	0.731	0.4
AR: 0.7 / CR: 0.25	Offset	3	7	0.773	0.735	-0.031	0.013	0.046	0.032	0.756	0.492
AR: 0.7 / CR: 0.25	Offset	6	3	0.775	0.423	-0.027	-0.708	0.044	0.082	0.721	0
AR: 0.7 / CR: 0.25	Offset	6	7	0.84	0.73	0.002	-0.479	0.032	0.104	0.704	0

Table S3.
Cross Regressive Parameter Outcomes for 100 Timepoint Conditions. N Items: Number of Items per State; N Responses: Number of Response Categories per Item.

AR/CR Parameters	Spread	N Items	N Responses	Median Bias		Median Slice SE		Coverage	
				GRM	Linear	GRM	Linear	GRM	Linear
AR: 0.3 / CR: 0	Equal	3	3	-0.005	0.42	0.16	0.11	0.823	0.204
AR: 0.3 / CR: 0	Equal	3	7	0.008	0.371	0.164	0.093	0.82	0.143
AR: 0.3 / CR: 0	Equal	6	3	0.008	0.489	0.116	0.205	0.778	0.411
AR: 0.3 / CR: 0	Equal	6	7	0.003	0.395	0.114	0.155	0.803	0.36
AR: 0.3 / CR: 0	Offset	3	3	-0.024	0.428	0.18	0.115	0.8	0.193
AR: 0.3 / CR: 0	Offset	3	7	-0.02	0.381	0.162	0.092	0.85	0.147
AR: 0.3 / CR: 0	Offset	6	3	0.015	0.367	0.147	0.204	0.814	0.337
AR: 0.3 / CR: 0	Offset	6	7	0.008	0.424	0.109	0.172	0.759	0.388
AR: 0.3 / CR: 0.25	Equal	3	3	0.047	0.316	0.164	0.119	0.753	0.365
AR: 0.3 / CR: 0.25	Equal	3	7	0.035	0.323	0.158	0.108	0.823	0.324
AR: 0.3 / CR: 0.25	Equal	6	3	0.016	0.279	0.113	0.213	0.783	0.548
AR: 0.3 / CR: 0.25	Equal	6	7	-0.011	0.244	0.112	0.153	0.774	0.591
AR: 0.3 / CR: 0.25	Offset	3	3	0.029	0.264	0.183	0.124	0.763	0.431
AR: 0.3 / CR: 0.25	Offset	3	7	0.014	0.305	0.165	0.102	0.827	0.367
AR: 0.3 / CR: 0.25	Offset	6	3	-0.011	0.185	0.146	0.227	0.777	0.385
AR: 0.3 / CR: 0.25	Offset	6	7	0.014	0.274	0.11	0.198	0.734	0.63
AR: 0.7 / CR: 0	Equal	3	3	0	0.273	0.11	0.078	0.743	0.157
AR: 0.7 / CR: 0	Equal	3	7	0	0.248	0.098	0.061	0.783	0.133
AR: 0.7 / CR: 0	Equal	6	3	0.006	0.342	0.081	0.197	0.736	0.492
AR: 0.7 / CR: 0	Equal	6	7	-0.017	0.225	0.077	0.117	0.779	0.559
AR: 0.7 / CR: 0	Offset	3	3	-0.007	0.289	0.121	0.084	0.753	0.213
AR: 0.7 / CR: 0	Offset	3	7	-0.013	0.252	0.112	0.062	0.829	0.151
AR: 0.7 / CR: 0	Offset	6	3	0.006	0.318	0.094	0.211	0.736	0.354
AR: 0.7 / CR: 0	Offset	6	7	0.002	0.353	0.071	0.164	0.698	0.565
AR: 0.7 / CR: 0.25	Equal	3	3	0.053	0.202	0.115	0.118	0.783	0.533
AR: 0.7 / CR: 0.25	Equal	3	7	0.067	0.204	0.113	0.095	0.823	0.43
AR: 0.7 / CR: 0.25	Equal	6	3	0.055	0.227	0.077	0.23	0.695	0.617
AR: 0.7 / CR: 0.25	Equal	6	7	0.027	0.164	0.075	0.165	0.716	0.732
AR: 0.7 / CR: 0.25	Offset	3	3	0.074	0.231	0.127	0.124	0.795	0.456
AR: 0.7 / CR: 0.25	Offset	3	7	0.075	0.246	0.106	0.106	0.776	0.425
AR: 0.7 / CR: 0.25	Offset	6	3	0.062	0.281	0.095	0.218	0.705	0.337
AR: 0.7 / CR: 0.25	Offset	6	7	0.018	0.216	0.072	0.201	0.712	0.667

Table S4.
Cross Regressive Parameter Outcomes for 500 Timepoint Conditions. N Items: Number of Items per State; N Responses: Number of Response Categories per Item.

AR/CR Parameters	Spread	N Items	N Responses	Median Bias		Median Slice SE		Coverage	
				GRM	Linear	GRM	Linear	GRM	Linear
AR: 0.3 / CR: 0	Equal	3	3	-0.006	0.331	0.065	0.043	0.715	0.037
AR: 0.3 / CR: 0	Equal	3	7	-0.002	0.365	0.06	0.036	0.721	0.01
AR: 0.3 / CR: 0	Equal	6	3	0.004	0.476	0.048	0.072	0.764	0.111
AR: 0.3 / CR: 0	Equal	6	7	-0.001	0.434	0.045	0.059	0.708	0.05
AR: 0.3 / CR: 0	Offset	3	3	-0.005	0.312	0.069	0.045	0.762	0.051
AR: 0.3 / CR: 0	Offset	3	7	0.001	0.353	0.06	0.036	0.74	0.01
AR: 0.3 / CR: 0	Offset	6	3	0.008	0.292	0.055	0.079	0.758	0
AR: 0.3 / CR: 0	Offset	6	7	0.003	0.465	0.041	0.062	0.693	0.073
AR: 0.3 / CR: 0.25	Equal	3	3	-0.012	0.167	0.064	0.05	0.727	0.168
AR: 0.3 / CR: 0.25	Equal	3	7	0.005	0.266	0.059	0.041	0.765	0.124
AR: 0.3 / CR: 0.25	Equal	6	3	-0.004	0.054	0.046	0.075	0.72	0.198
AR: 0.3 / CR: 0.25	Equal	6	7	-0.016	0.284	0.043	0.06	0.697	0.157
AR: 0.3 / CR: 0.25	Offset	3	3	0.005	0.228	0.07	0.051	0.811	0.179
AR: 0.3 / CR: 0.25	Offset	3	7	0.006	0.285	0.058	0.046	0.726	0.144
AR: 0.3 / CR: 0.25	Offset	6	3	0.014	-0.109	0.057	0.065	0.748	0
AR: 0.3 / CR: 0.25	Offset	6	7	-0.009	0.274	0.042	0.068	0.629	0.169
AR: 0.7 / CR: 0	Equal	3	3	-0.003	0.165	0.036	0.02	0.742	0.03
AR: 0.7 / CR: 0	Equal	3	7	0.002	0.181	0.034	0.018	0.796	0.007
AR: 0.7 / CR: 0	Equal	6	3	0	0.256	0.03	0.069	0.711	0
AR: 0.7 / CR: 0	Equal	6	7	-0.003	0.278	0.028	0.055	0.69	0.066
AR: 0.7 / CR: 0	Offset	3	3	-0.003	0.16	0.037	0.024	0.765	0.075
AR: 0.7 / CR: 0	Offset	3	7	-0.004	0.165	0.035	0.016	0.773	0.003
AR: 0.7 / CR: 0	Offset	6	3	0.003	0.205	0.031	0.066	0.71	0.125
AR: 0.7 / CR: 0	Offset	6	7	0.004	0.319	0.027	0.065	0.709	0.126
AR: 0.7 / CR: 0.25	Equal	3	3	0.011	0.096	0.038	0.041	0.747	0.191
AR: 0.7 / CR: 0.25	Equal	3	7	0.018	0.13	0.036	0.033	0.714	0.182
AR: 0.7 / CR: 0.25	Equal	6	3	-0.003	-0.01	0.028	0.076	0.729	0.188
AR: 0.7 / CR: 0.25	Equal	6	7	0.003	0.193	0.025	0.063	0.615	0.288
AR: 0.7 / CR: 0.25	Offset	3	3	0.022	0.114	0.041	0.04	0.731	0.115
AR: 0.7 / CR: 0.25	Offset	3	7	0.012	0.167	0.035	0.037	0.67	0.175
AR: 0.7 / CR: 0.25	Offset	6	3	0.013	-0.054	0.032	0.077	0.711	0.084
AR: 0.7 / CR: 0.25	Offset	6	7	0.001	0.232	0.025	0.071	0.577	0.259

2. Slice Likelihood Standard Error Figures

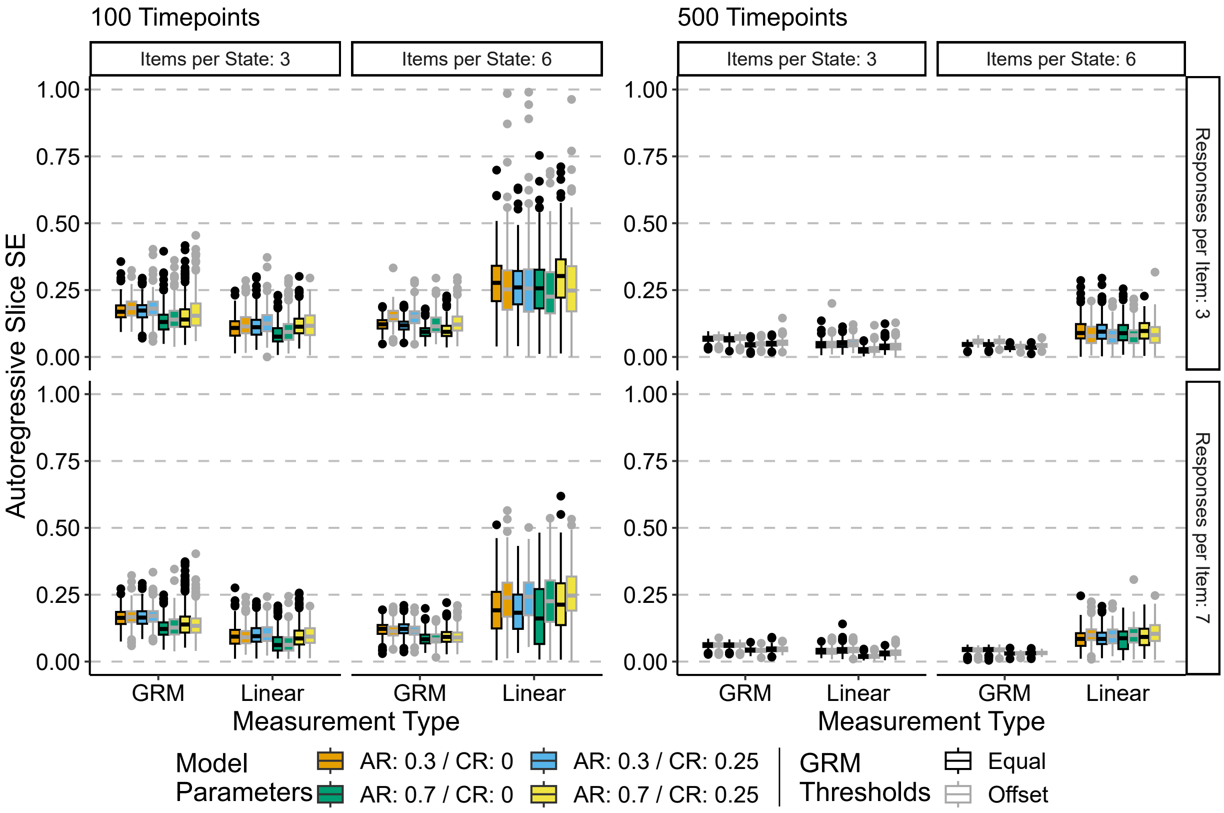

FIGURE S1.
Autoregressive Parameter Slice SE. Central black line on boxplot denotes median, box denotes 25% − 75% interquartile range, whiskers denote $1.5 \times IQR$. AR refers to the autoregressive parameter while CR refers to the cross-regressive parameter.

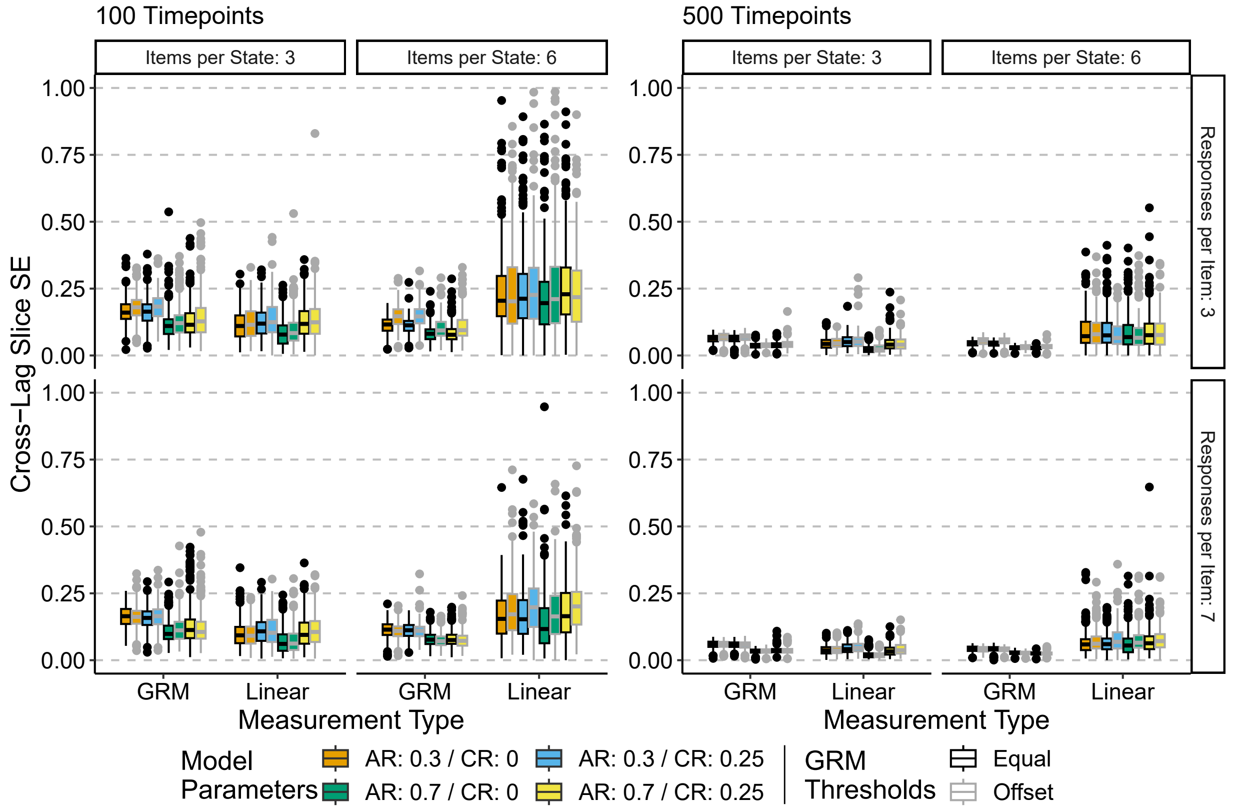

FIGURE S2.
Cross-regressive Slice SE. Central black line on boxplot denotes median, box denotes 25% − 75% interquartile range, whiskers denote 1.5 × IQR. AR refers to the autoregressive parameter while CR refers to the cross-regressive parameter.
